# Synthesis of Nickel Spinel Ferrites Nanoparticles Coated with Thermally Reduced Graphene Oxide for EMI Shielding in the Microwave, UV, and NIR Regions

**DOI:** 10.3390/polym13193316

**Published:** 2021-09-28

**Authors:** Asim Mansha, Khadija Zubair, Zulfiqar Ahmad Rehan, H. M. Fayzan Shakir, Talha Javed, Rubab Shabbir, Syed Khalid Mustafa, Freddy Mora-Poblete, Jing-Ru Zhou, Uttam Kumar, Mohammad S. Al-Harbi, Mohamed M. Hassan

**Affiliations:** 1 Department of Chemistry, Government College University, Faisalabad 38000, Pakistan; mansha.asim@gmail.com; 2 School of Materials Science and Engineering, Northwestern Polytechnical University, Xi’an 710000, China; Khadijazubair65@mail.nwpu.edu.cn; 3 Department of Materials, School of Engineering and Technology, National Textile University, Faisalabad 37610, Pakistan; 4 College of Agriculture, Fujian Agriculture and Forestry University, Fuzhou 350002, China; mtahaj@fafu.edu.cn (T.J.); rubabshabbir28@gmail.com (R.S.); zjr_fafu2019@126.com (J.-R.Z.); 5 Department of Agronomy, University of Agriculture, Faisalabad 38000, Pakistan; 6 Seed Science and Technology, University of Agriculture, Faisalabad 38000, Pakistan; 7 Department of Chemistry, Faculty of Science, University of Tabuk, Tabuk 71491, Saudi Arabia; syed.pes@gmail.com; 8 Institute of Biological Sciences, Campus Talca, Universidad de Talca, Talca 3465548, Chile; morapoblete@gmail.com; 9 College of Plant Protection, Fujian Agriculture and Forestry University, Fuzhou 350002, China; uttam5454@gmail.com; 10 Department of Biology, College of Science, Taif University, P.O. Box 11099, Taif 21944, Saudi Arabia; mharbi@tu.edu.sa (M.S.A.-H.); khyate_99@yahoo.com (M.M.H.)

**Keywords:** nanoparticles, nickel spinal ferrites, thermally reduced graphene oxide, nanocomposites, polymeric nanocomposites, EMI shielding

## Abstract

The co-precipitation and in situ modified Hummers’ method was used to synthesize Nickel Spinal Ferrites (NiFe) nanoparticles and NiFe coated with Thermally Reduced Graphene Oxide (TRGO) (NiFe-TRGO) nanoparticles, respectively. By using polyvinyl chloride (PVC), tetrahydrofuran (THF), and NiFe-TRGO, the nanocomposite film was synthesized using the solution casting technique with a thickness of 0.12–0.13 mm. Improved electromagnetic interference shielding efficiency was obtained in the 0.1–20 GHz frequency range. The initial assessment was done through XRD for the confirmation of the successful fabrication of nanoparticles and DC conductivity. The microstructure was analyzed with scanning electron microscopy. The EMI shielding was observed by incorporating a filler amount varying from 5 wt.% to 40 wt.% in three different frequency regions: microwave region (0.1 to 20 GHz), near-infrared (NIR) (700–2500 nm), and ultraviolet (UV) (200–400 nm). A maximum attenuation of 65 dB was observed with a 40% concentration of NiFe-TRGO in nanocomposite film.

## 1. Introduction

The electronic complications of devices or electronic systems due to greater density packing increases the interference of electromagnetic (EM) radiation (EMR) [1,2]. Wearable and portable electronic systems such as mobile phones, smartwatches, and health-monitoring systems are becoming a need in many individuals’ life because of their instant development with multi-functionality and efficiency [3,4,5]. The production of heat from advanced electronic materials has a harmful effect on the functions of the electronic system as well as on human health [6,7]. These complications are creating the undesired emission of radiation or electronic pollution known as electromagnetic interference (EMI). The EM noise can be produced by an EMR of any frequency range. Interference in the radio frequency range is a novel form of EMI, and some other kinds of interference are microwave, X-ray, light, cosmic rays, and heat energy. This EMI involves the EM pulses as well as the explosion of EM energy such as nuclear explosion, lightning, and electrostatic discharge [8]. EMI shielding is a technique used to attenuate the emission of radiation. These shielding nanomaterials have been extensively utilized in several fields of the communication system as well as aerospace systems depending on their ability to attenuate interference radiation [9,10]. 

Lorentz’s force law states the mechanism of EMI shielding occurs when EMR interacts on the shielding surface and the electrons of the shielding material interact with the incident EMR that produces the EM field. The direction of the induced field is the reverse of the incident EM waves. The energy of the incident EM radiation is reduced by the induced EMR. Three parameters contribute to the mechanism of shielding: absorption, reflection, and multiple reflections [11]. These three kinds of shielding are based on their shielding principles, such as magnetic field shielding, electric field shielding, and the EM field. The basic purpose of shielding is to block the entrance of EM waves [12]. The main principle mechanism of electrostatic shielding involves the field of external electrostatic discharge in which the charge of the conductor surface is reorganized to attain the field until the conductor reaches zero strength. Metals provide outstanding electrostatic shielding for EMI. The principle of magnetic field shielding is based on blocking off radiation at the external magnetic field, the low-frequency field. As the permeability value of the nanomaterials increases, the magnetic resistance will decrease, creating a magnetic flux loop toward the magnetic resistance. Ferromagnetic materials are excellent nanomaterials for magnetic field shielding at low frequencies [13,14]. EMI shielding at a high-frequency range requires the magnetic field to always be reversed to the original magnetic field produced by the current in excellent conductors [15,16,17,18,19,20,21,22]. This outstanding EMI shielding evaluates the usage of metals as well as magnetic nanomaterials to attenuate electric and magnetic field radiation [23,24,25,26,27,28,29,30]. Zubair et al. synthesized thermally reduced graphene oxide (TRGO) and barium ferrites (BaFe) nanocomposites to improve the shielding efficiency solution casting method. A shielding efficiency of 62 dB was obtained at 12.5 GHz with improved conductivity values of 4.8 × 10^−5^ [31]. In another work, Shakir et al., synthesized NiFe to evaluate the effect on EMI shielding. A shielding efficiency of 35 dB was obtained at the 0–20 GHz frequency range with polystyrene/polyaniline/nickel-ferrites (PS/PANI/NiFe) nanocomposites [32]. In another work, a shielding efficiency of 29 dB was obtained in the 9–18 GHz frequency range with a TRGO/polymer [20].

A nanocomposite based on a polyvinyl chloride matrix was prepared in this research. The reinforcing material was prepared by coating nickel ferrite particles with thermally reduced graphene oxide (TRGO) by in situ modified Hummers’ method. The initial assessment was carried out through XRD and DC conductivity. The EMI shielding was observed with the incorporation of fillers from 5% to 40% in three different regions: microwave (0.1 to 20 GHz), near-infrared (NIR) (700–2500 nm), and ultraviolet (UV) (200–400 nm). Thermally reduced Graphene Oxide (TRGO) was used for its high electrical conductivity and good dispersibility in the polymer matrix. Nickel Ferrites (NiFe) have poor dispersibility but better EMI shielding properties. TRGO was coated onto NiFe nanoparticles to increase its dispersibility in the polymer matrix. With this mechanism, a novel nanoparticle was produced in which NiFe nanoparticles were covered with TRGO making it the core with magnetic properties and a surface with high electrical conductivity.

## 2. Materials and Methods

Nickel Ferrites were prepared in previous work using the co-precipitation method. Graphite powder, phosphoric acid (H_3_PO_4_) (98%), sulfuric acid (H_2_SO_4_) (96%), and hydrogen peroxide (H_2_O_2_) (30%) tetrahydrofuran (THF), were all purchased from Sigma-Aldrich, Burlington, VT, USA, and other materials such as potassium permanganate (KMnO_4_), deionized water (H_2_O), and polyvinylchloride (PVC) were purchased from the Engro Group of Industries Pakistan.

### 2.1. Preparation of Nickel Ferrite (NiFe)

The nickel ferrite (NiFe) nanoparticles were synthesized by using the co-precipitation technique. Three 200 mL solutions of Nickel nitrate (0.1 M), iron nitrate (0.2 M), and sodium hydroxide (3 M) were formed separately. Nickel nitrate and iron nitrate solution were mixed together, and sodium hydroxide solution was added afterward dropwise. After mixing, the solution was stirred continuously for 1 h at 80 °C. Then, stirring was carried out at room temperature for 4 h. The mixture was washed several times after stirring to attain 7 pH. The precipitates were dried at 100 °C for 1 h after centrifugation. The precipitates were calcinated for 6 h at 850 °C to obtain the NiFe nanoparticles. 

### 2.2. Preparation of Nickel Ferrite (NiFe) Coated with Thermally Reduced Graphene Oxide (TRGO) Nanoparticles (TRGO-NiFe)

For the synthesis of NiFe-TRGO, NiFe (3 g) nanoparticles were mixed with graphite (3 g), H_2_SO_4_ (360 mL), KMnO_4_ (18 g), and H_3_PO_4_ (40 mL) with continuous stirring for 24 h at 50 °C. In all, 20 mL of 30% solution of H_2_O_2_ was added to the solution mixture to stop the reaction by reacting with unreacted KMnO_4_. To maintain the temperature of the reaction, the ice of deionized water (400 mL) was added, and it formed the light brown solution of the NiFe-GO (Graphene Oxide) which was then centrifuged. To reduce the NiFe-GO, it was kept in the oven for 72 h at 200 °C. The NiFe-TRGO nanoparticles were obtained. 

### 2.3. Preparation of Nanocomposite Films

The nanocomposite film was synthesized by using PVC as a matrix of film in which NiFe-TRGO was used as a nanofiller. PVC and NiFe-TRGO were mixed in 50 mL of THF in required concentrations and sonicate for 2 h. THF was only used as a solvent and evaporated in the drying step. The stirring was carried out after sonication for 24 h which is then poured into the petri dish. The mixture was first dried at room temperature for 5 h then in the oven at 50 °C. The final PVC/NiFe-TRGO nanocomposite film was obtained. The composition of each prepared nanocomposite film is given Table 1.

### 2.4. Characterization

#### 2.4.1. X-ray Diffraction

D8 Discover of BRUKER (Billerica, MA, USA) was used for the x-ray analyses from 2θ 10° to 70° with an increment of 0.05° and stay time of 1 s. The obtained graphs are shown in Section 3.1.

#### 2.4.2. Scanning Electron Microscopy

“JEOL-instrument JSM-6490A” was used to capture a micrograph of nanocomposites films. Films were dipped in liquid nitrogen and broken to expose a fresh unaltered cross-sectional area. SEM images of cross-sectional are shown in the Results and Discussion Section of this article. 10 KV voltage and LVSTD detector were used. Nanocomposite films were coated with gold by sputter coater with a thickness of 5 to 10 A^0^.

#### 2.4.3. UV/Vis/NIR Spectroscopy

“Lambda 950, Perkin Elmer” were used to evaluate the transmission of UV/Vis/NIR waves through the nanocomposite films in the whole UV/Vis/NIR region of wavelengths from 200 to 2500 nm.

#### 2.4.4. DC Conductivity

Keithley 2450 was used to measure the DC conductivity of prepared nanocomposite films. Probes were placed at a distance of 5 mm. The current was measured by using an ammeter and applying 5 V of voltage. The DC conductivity was then calculated by using the value of resistance and resistivity with the following relationship:

(1) $$R=\frac{V}{I}$$

(2) $$\rho =\frac{RA}{L}$$

(3) $$\sigma =\frac{1}{\rho }$$

in which *R*, *I*, and *V* represent the resistance, current, and voltage, respectively. *A* and *L* represent the area of cross-section (0.5 mm × 10 mm) and length (10 mm), respectively, while *ρ* and *σ* represent the value of conductivity and resistivity, respectively. 

#### 2.4.5. Vector Network Analyzer

Rohde & Schwarz’ ZNB20 (Munich, Germany) was used in this research work. Samples were cut according to the required dimension by a highly precise laser cutter. A donut shape sample was required for this test. A circular sample with an outer diameter of 7 mm with a 3 mm hole in the center. Scattering parameters, capacitance, and dissipation factor were obtained from VNA. By using relationships given in the Results and Discussion Section, EMI shielding was both calculated by capacitance and dissipation factor and measured by scattering parameters. 

## 3. Results and Discussion

### 3.1. X-ray Diffraction

**Figure 1:**
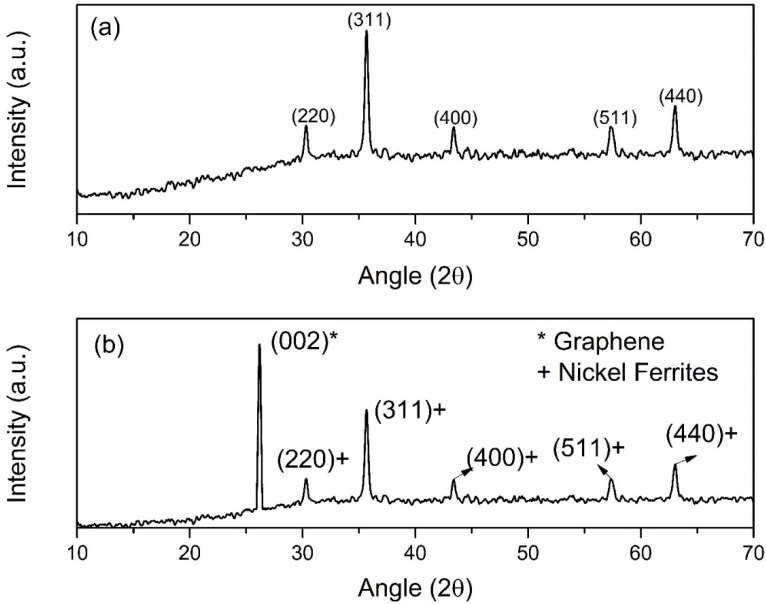
XRD pattern of (**a**) NiFe nanoparticles, (**b**) NiFe-TRGO nanoparticles.

The structure of NiFe and NiFe-TRGO nanoparticles was evaluated by X-ray diffraction with diffractions of 10–70°. The XRD pattern of synthesized NiFe and NiFe-TRGO nanoparticles is presented in Figure 1. The formation of the XRD pattern represents the single-layered structure. These diffraction peaks indicated the formation of particles in the nanometer range which resulted in an increase in temperature of the sample. The size of NiFe nanoparticles also increased and led to crystal growth of the NiFe nanoparticles. The reflection planes were observed at (220), (311), (400), (511), and (440) at an angle of 30°, 36°, 44°, 58°, and 63°, respectively [33]. It was observed in the previous research that prepared nanoparticles are spherical in nature and have a diameter of almost 50–60 nm [32]. Figure 1b indicates the presence of the TRGO with its characteristic peaks at the reflection plane of (002). The TRGO was also prepared separately in previous work without the addition of NiFe nanoparticles. It was found to have a 10–15 nm thick layer. Additionally, the same XRD pattern was observed [21].

### 3.2. Scanning Electron Microscopy

**Figure 2:**
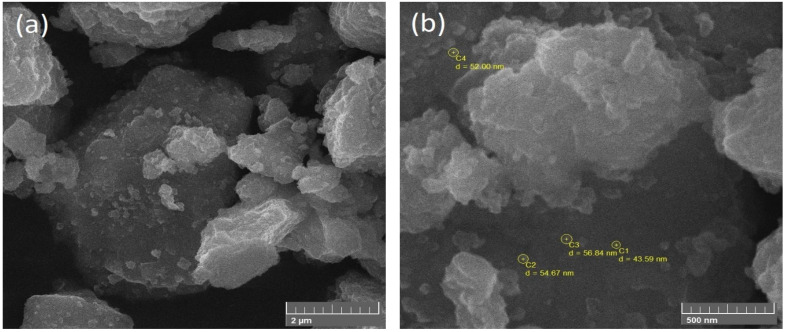
SEM Images of NiFe nanoparticles on (**a**) low and (**b**) high resolution.

**Figure 3:**
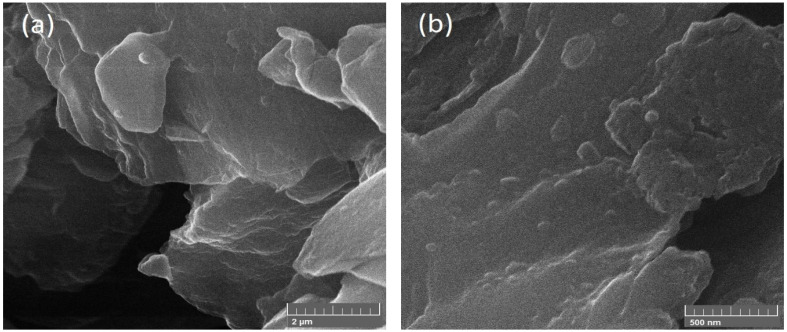
SEM Images of NiFe-TRGO nanoparticles on (**a**) low and (**b**) high resolution.

**Figure 4:**
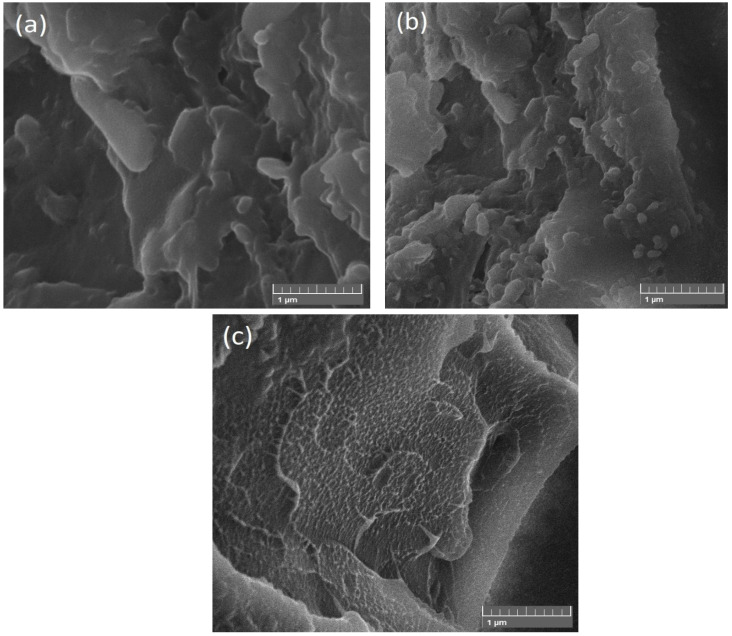
SEM Images of (**a**) S1, (**b**) S2, and (**c**) S4.

**Figure 5:**
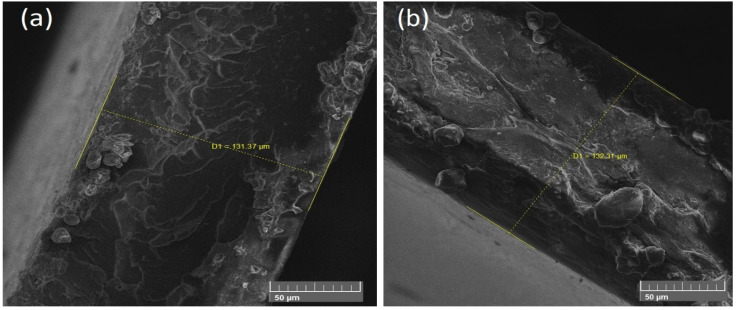
Measured thickness of nanocomposite films by SEM (**a**) and (**b**).

To investigate the shape and size of nanoparticles, SEM (JEOL-instrument JSM-6490A) was used. From SEM images, it was clear that NiFe nanoparticles have a spherical structure rather than a cube, and the average diameter of spherical particles was found out to be 50 nm. Figure 2 represents the NiFe nanoparticles at the low and high-resolution angles which indicate a spherical structure and 50 nm diameter. Figure 3 represents the NiFe-TRGO nanoparticles at low and high resolution that clearly show the interconnected network between NiFe and TRGO nanoparticles. It is also evident that NiFe nanoparticles are attached onto the TRGO sheets. There were several NiFe nanoparticles placed onto the sheet of TRGO as clearly visible in Figure 3b. For microstructure analysis of NiFe-TRGO nanocomposites film, the samples were first dipped in liquid nitrogen and then broken into pieces to obtain the fine edges of the film. Figure 4 represents the analysis of NiFe-TRGO nanocomposites film with smooth surfaces having a densely interconnected network of nanoparticles. It is observed that the density of interconnected network structures increases with an increase in filler amount from 5 wt% to 40 wt%. The thickness of nanocomposite films was also measured by SEM and found to be approximately 113 µm as shown in Figure 5.

### 3.3. DC Conductivity

**Figure 6:**
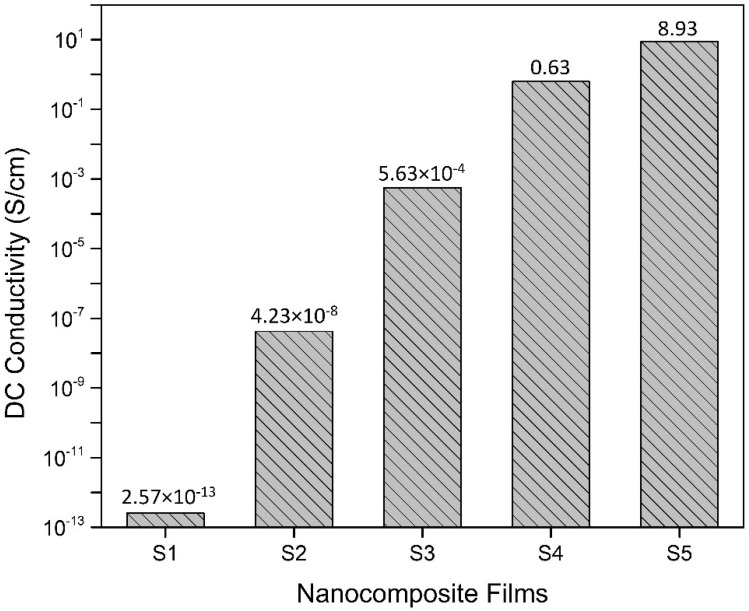
DC conductivity values of prepared nanocomposite films.

The values of DC conductivity are represented in Figure 6. S1 has a conductivity value of 2.57 × 10^−13^ S/cm which confirms the insulative nature of PVC [19]. S2 has an increased conductivity value of 4.23 × 10^−8^ S/cm that is attributed to the presence of electrically conductive nanoparticles. S3 and S4 have conductivity values of 5.63 × 10^−^^4^ S/cm and 0.63 S/cm that show the large increase in the conductivity due to the increase in the concentration of NiFe-TRGO nanoparticles. The S5 sample has the greatest value of DC conductivity at 8.93 S/cm with a 40% concentration of NiFe-TRGO. The graphical representation indicates the improved value of DC conductivity with an increasing concentration of nanofillers. The increase in electrical conductivity is caused by the formation of an interconnected network structure of nanoparticles inside the polymer matrix. As the concentration of nanoparticles keeps increasing, the structure density also increases, resulting in enhanced electrical conduct. The formation of a mature interconnected network structure is also seen and confirmed by SEM in Figure 4c. 

### 3.4. EMI Shielding in UV/Vis/NIR Region

**Figure 7:**
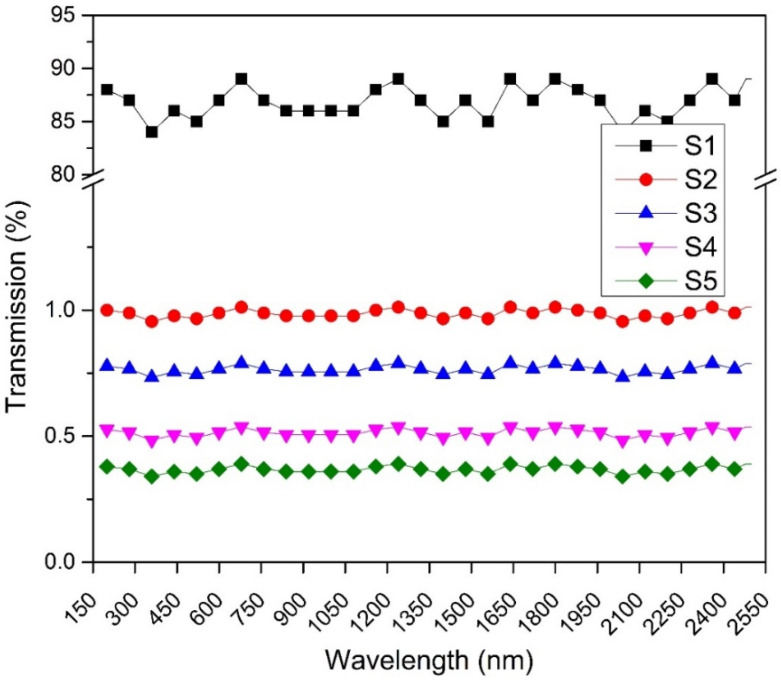
UV/Vis/NIR transmissions.

Figure 7 shows the attenuation of radiation through the nanocomposite film. For pure PVC, the transmission radiations are about 80–90% in S1 [18]. By adding 5% NiFe-TRGO nanoparticles, the transmission of radiation decreases to almost 1% in S2 and is consistent with transmission of microwave radiation 10 dB resulting in 90% shielding and 1% transmission. By adding 15% NiFe-TRGO nanoparticles, transmission decreased to <1% in S3. By adding 30% and 40% in S4 and S5, respectively, the transmission reached near 0%. These results proved NiFe nanoparticles are strongly absorptive and TRGO particles are strongly reflective and have the ability to attenuate the radiation in the NIR region.

### 3.5. EMI Shielding in the Microwave Region Calculated through Impedance Parameters

The EMI shielding in this research work was calculated as follows:

*SE*_*T*_ = *SE*_*R*_ + *SE*_*A*_ + *SE*_*MR*_ (4)

where *SE_T_* is total shielding effectiveness, *SE_R_* is shielding through reflection, *SE_A_* shielding through absorption, and *SE_MR_* shielding through multiple reflections. As the composite film prepared was very thin, multiple reflections were negligible. Capacitance and dissipation factor were measured by Vector Network Analyzer in the microwave region (0.1 GHz to 20 GHz), and SER and SEA were calculated by the following formulas:

(5) $$SE_{A}=8.8\;\alpha l$$

(6) $$SE_{R}=20\log\frac{{\left|1+n\right|}^{2}}{4n}$$

(7) $$\alpha =\frac{2\pi }{\lambda }\sqrt{\frac{\varepsilon^{\prime }\sqrt{1+tan^{2}\delta \;}}{2}\;\;}$$

(8) $$n=\sqrt{\frac{\varepsilon^{\prime }\sqrt{1+tan^{2}\delta \pm 1\;}}{2}}+i\sqrt{\frac{\varepsilon^{\prime }\sqrt{1+tan^{2}\delta \pm 1\;}}{2}}$$

(9) $$\varepsilon^{\prime }=Cl÷A\varepsilon_{o}$$

where “l” is the thickness of film, *ε*′ is dielectric constant, *A* is the area of film, *λ* wavelength, *C* is capacitance, *ε*_o_ is the dielectric permittivity of free space, and *tanδ* is the dissipation factor [34]. 

**Figure 8:**
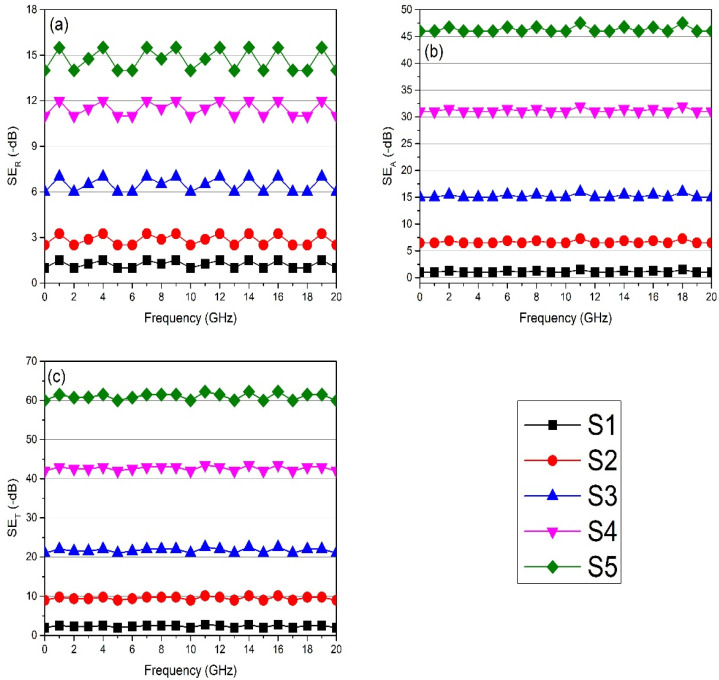
Calculated EMI shielding of prepared nanocomposite films through impedance parameters (**a**) *SE_R_*, (**b**) *SE_A_*, and (**c**) *SE_T_*.

Figure 8 shows the relation between shielding effectiveness due to reflection SER and frequency calculated by the above-mentioned equations. Pure PVC has very little reflection, up to 2 dB which decreased gradually to 0 dB. By adding 5% NiFe-TRGO, it showed 12 dB SET, as an interconnected network structure of nanoparticles is formed that provides electrical conductivity. With the addition of 15% nanofiller, it showed 20 dB. When concentration was 30%, it gave 42 dB and at 40% 62 dB. The shielding effectiveness improved by enhancing the concentration of NiFe-TRGO which improved the conductivity value that ultimately enhanced the shielding efficiencies. The absorption phenomenon was dominant because of the presence of ferromagnetic nanoparticles.

### 3.6. EMI Shielding in the Microwave Region Measured through Scattering Parameters

The characteristic efficiency of EMI shielding was also measured by using the vector network analyzer. A rectangular strip (10 mm × 5 mm) of nanocomposite film was used to measure the efficiency. It is clear from EM theory that the interaction of the EM wave with shielding materials, will either reflect, absorb, or transmit the wave [35]. The scattering parameters were evaluated by VNA. Permeability and permittivity parameters are also widely utilized for understanding the shielding mechanism in the frequency range of the microwave region. The EMI shielding efficiency was measured by scattering parameter of reflection, absorption, and multiple reflections with vector network analyzer which is stated as [36]:

(10) $$SE_{T\;\;}=SE_{A\;\;\;}+SE_{R\;}+SE_{MR}$$

(11) $$SE_{T}=10log\frac{1}{{\left|S_{12}\right|}^{2}}=10log\frac{1}{{\left|S_{21}\right|}^{2}}\;\;\;$$

(12) $$SE_{R\;}=10log_{10\;}\left(\frac{1}{\left(1-S_{11}^{2}\right)}\right)$$

(13) $$SE_{A}=10log_{10}\left(\frac{\left(1-S_{11}^{2}\right)}{S_{12}^{2}}\right)$$

The scattering parameter represents *S*_11_ as the coefficient of forwarding reflection, *S*_12_ as the coefficient of reverse transmittance, and *S*_21_ represents the coefficient of forwarding transmission. The power of transmission and reflection characterizes the scattering parameter. The EMI shielding effectiveness was observed and calculated in dB by using the following relation.

**Figure 9:**
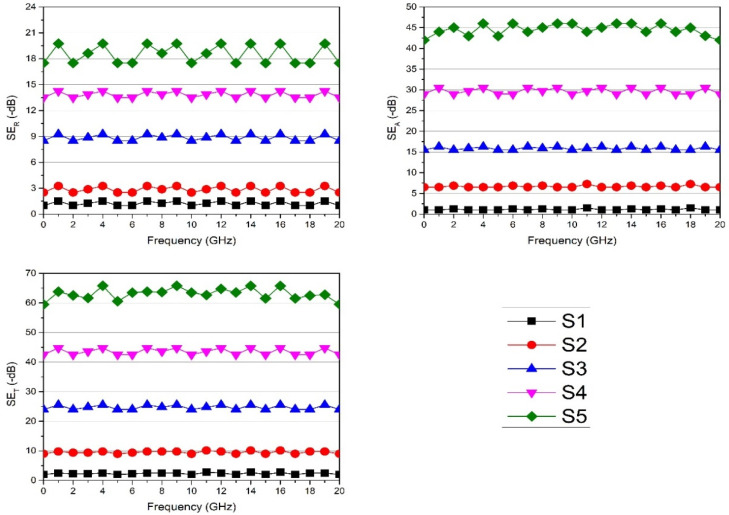
Measured EMI shielding of prepared nanocomposite films through VNA (**a**) *SE_R_*, (**b**) *SE_A_*, and (**c**) *SE_T_*.

The S1 sample had almost zero shielding efficiency in both SEA and SER, and that indicates the insulative nature of the materials. The S2 sample had an efficiency of 10 dB at the 0–20 GHz frequency with the addition of 5% NiFe-TRGO nanoparticles. By increasing the concentration of NiFe-TRGO up to 15% the EMI SE reached 23 dB in the S3 sample. The addition of NiFe-TRGO with 30% and 40% concentration led to shielding efficiency at 45 dB and 65 dB with a minimum film thickness of 0.13 mm for the S4 and S5 samples, respectively. The shielding effectiveness improved by enhancing the concentration of NiFe-TRGO which improved the conductivity value that ultimately enhanced the shielding efficiencies. The absorption phenomenon was dominant because of the presence of ferromagnetic nanoparticles. EMI shielding of prepared nanocomposite films measured through VNA is represented in Figure 9. EMI shielding of NiFe and TRGO was also tested separately in previous work. NiFe has poor dispersibility in a polymer matrix and gave almost 30 dB shielding effectiveness at 20 wt.% loading, while TRGO has good dispersibility and gave almost 35 dB shielding effectiveness with 5 wt.% loading [30,31,32].

## 4. Conclusions

By using NiFe-TRGO nanoparticles and Polyvinyl chloride (PVC), the nanocomposite films were synthesized by varying the concentration of NiFe-TRGO nanoparticles through the solution casting technique. A minimum thickness of 0.12–0.13 mm was achieved, which makes it novel for commercial use. The structural analysis was performed with X-ray diffraction and a scanning electron microscope. The present work found that by increasing the concentration of NiFe-TRGO, the value of DC conductivity increases because electrically conductive nanoparticles and transmission value decreased to *a* <1 value for Samples S2–S5. The EMI shielding value was observed with an impedance analyzer and vector network analyzer. The maximum shielding efficiency obtained was 65 dB by the S5 sample at a frequency range of 0–20 GHz. The NiFe-TRGO nanoparticles provided a reflecting and absorption site to enhance the shielding effectiveness. The synthesized nanocomposite proved to be very beneficial on a commercial scale.

## Figures and Tables

**Table 1:** The composition of nanocomposites film of PVC/NiFe-TRGO.

Sr. No.	Sample Name	PVC (wt%)	NiFe-TRGO (wt%)	Nanocomposite Film Thickness (µm)
1	S1	100	-	131
2	S2	95	5	133
3	S3	85	15	132
4	S4	70	30	130
5	S5	60	40	129

## Data Availability

Not applicable.
